# Unraveling the cytokine profile in acute Chagas disease in the Brazilian Amazon: Insights before and after treatment

**DOI:** 10.1371/journal.pntd.0013942

**Published:** 2026-01-30

**Authors:** Jessica Vanina Ortiz, Débora Raysa Teixeira de Sousa, Fernanda Gallinaro Pessoa, Marry Beatriz Matni, Gabriela Maciel Alencar, Nádelly Karoline Martins Derze, Alba Regina Jorge Brandão, Vânia Mairi Nauê, Elsa Isela Guevara Moctezuma, Orlando Ribeiro, Félix José Alvarez Ramires, Matheus Martins Monteiro, Kátia do Nascimento Couceiro, Maria das Graças Vale Barbosa Guerra, Jorge Augusto de Oliveira Guerra, Fábio Fernandes, João Marcos Bemfica Barbosa Ferreira

**Affiliations:** 1 Programa de Pós-Graduação em Medicina Tropical, Universidade do Estado do Amazonas, Manaus, Brazil; 2 Fundação de Medicina Tropical Dr. Heitor Vieira Dourado, Manaus, Brazil; 3 Instituto do Coração de São Paulo – Universidade de São Paulo, São Paulo, Brazil; 4 Universidade Federal do Amazonas, Manaus, Brazil; 5 Hospital Beneficência Portuguesa de São Paulo, São Paulo, Brazil; 6 Universidade do Estado do Amazonas, Manaus, Brazil; Tulane University School of Public Health and Tropical Medicine, UNITED STATES OF AMERICA

## Abstract

Chagas disease (CD) is a neglected anthropozoonosis caused by *Trypanosoma cruzi*, with the oral route standing out as the primary cause for the increase in acute cases in the Brazilian Amazon and a key factor in the increase of acute cases. Despite this, the immune response in affected individuals remains minimally explored in humans. This longitudinal study aimed to characterize the serum cytokine profile in patients with acute CD before and after treatment. Participants were recruited from several municipalities in the state of Amazonas, and individuals with chronic inflammatory diseases were excluded. The cytokines IL-1α, IL-2, IL-4, IL-5, IL-6, IL-10, IL-17α, TNF-α, and IFN-γ were analyzed. From 2017 to 2022, 35 patients and 14 controls were included (mean age 46.3 ± 17.8 years; 57.1% male; 74.3% from oral transmission outbreaks). Twenty-five patients (71.4%) were successfully followed up, despite logistical challenges. Elevated levels of all cytokines were found in acute-phase patients before treatment, indicating an effective immune activation. After an average of 12 months post-treatment, significant reductions were observed in IL-6 (p < 0.001), TNF-α (p = 0.011), IFN-γ (p < 0.001), and IL-10 (p = 0.015). IL-2 showed strong correlations with IL-1α and IL-17α in the acute phase (r = 0.91 and 0.95). Post-treatment cytokine profiles resembled those of the control group, suggesting treatment efficacy in modulating inflammation. IL-17α, IL-10, and IL-2 maintained an interesting pattern after treatment. Cardiac MRI revealed myocardial injury by late gadolinium enhancement (LGE) in 27.3% of pre-treatment and 33.3% of post-treatment patients. IL-1α, IL-17α, and IL-4 had moderate negative correlations (r = –0.36 to –0.67) with LGE. These results highlight the importance of monitoring the inflammatory response in the long term, as well as the observed cytokine modulation, which reinforces the hypothesis of not only a favorable acute immune response but also a sustained long-term clinical improvement following treatment.

## Author summary

Chagas disease is caused by *Trypanosoma cruzi* and remains a neglected tropical disease with increasing reports of acute cases in the Brazilian Amazon, largely due to oral transmission. Although the clinical aspects of acute CD are well described, the immune response during and after treatment is still poorly described in human studies. In this longitudinal study, we analyzed the serum cytokine profiles of patients diagnosed with acute CD in Amazonas, Brazil, before and after benznidazole treatment. Our results show that cytokine levels—particularly IL-6, TNF-α, IFN-γ, and IL-10—were significantly elevated during the acute phase and decreased after treatment, suggesting effective immune modulation. Interestingly, IL-2 strongly correlated with IL-1α and IL-17α during the acute phase, and the post-treatment cytokine profile resembled that of healthy controls. Cardiac MRI revealed myocardial injury by LGE in a subset of patients before and after treatment, highlighting the need for long-term follow-up. Certain cytokines, such as IL-1α, IL-17α, and IL-4, also showed negative correlations with cardiac fibrosis markers. This study emphasizes the value of monitoring immune responses beyond the acute phase; these findings also offer insights into the disease progression and support the hypothesis that early treatment not only controls infection but may also promote sustained clinical and immunological recovery.

## Introduction

Chagas disease (CD) is a worldwide recognized neglected tropical disease caused by *Trypanosoma cruzi,* a parasite that can be transmitted to humans through vectors of the Triatominae family, commonly known as kissing bugs [1]. In addition to vector-borne transmission, other significant routes include: blood transfusion, organ transplantation, laboratory accidents, vertical transmission, and through contaminated food, especially in the Amazon region [2]. Oral transmission is currently the main route of infection in acute cases, to which the state of Amazonas has reported 11 outbreaks due to the consumption of palm fruit juice from 2004 to 2024, with more than 100 acute cases [3].

During the acute phase, the immune response to the parasite triggers parasitemia control processes through innate immune cells, such as neutrophils, macrophages, and natural killer (NK) cells. However, the response in the chronic phase is mediated by lymphocytes, which play an important role in disease regression [4,5]. The cellular immune response in acute Chagas disease (ACD) has been little addressed in humans. Most studies during this acute phase are experimental with a murine animal model, in which the inflammatory profile demonstrates a balance between pro- and anti-inflammatory cytokines, an essential process for mediating the progression of CD, especially in the chronic phase, and their expression is associated with the morbidity of Chagas disease [6,7].

The inflammatory action is controlled by the expression of anti-inflammatory cytokines, such as IL-10 and TGF-β, which play critical roles in regulating the host’s immune response to *T. cruzi*. In chronic phase inflammatory lesions, whether in patients with Chagas cardiomyopathy (CCC) or indeterminate form, T cell activation is consistent, as is the presence of pro-inflammatory cytokines IL-4, IL-6, IL-12, TNF-α, and IFN-γ [8]. The increased expression of IL-6 cytokines, in addition to those that are part of the Th1 response, such as IFN-γ and TNF-α, is associated with CCC [8]. These cytokines are regulated by other anti-inflammatory Th2 response cytokines, such as IL-10 and IL-5, which are found in low concentrations [6].

The Th17 response has been shown to play an important role in the regulation of myocarditis induced by acute experimental infection with *T. cruzi*, suggesting control of resistance to infection [9]. After intense immunological activity during the acute phase, in which parasitemia is high, and attempts to keep the parasite under control during the indeterminate chronic phase, pathogenic autoimmune reactions by molecular mimicry and polyclonal activation occur in CCC. These mechanisms cause an increase in the production of pro-inflammatory cytokines (INF-γ and TNF-α) and a decrease in anti-inflammatory cytokines (IL-10), which supports the hypothesis that the progression to CCC develops an exacerbated Th1-type immune response [10,11].

Another relevant issue to point out is the effects of inflammatory activity identified by cardiac magnetic resonance (CMR) as the gold standard for quantification of cardiac fibrosis and edema, which are related to dysfunction, dilation, and ejection fraction of patients, and are correlated with the severity of the progression of CD [12]. Advanced imaging methods can aid in the study of the injury caused by the parasite in cardiac tissue. Post-inflammatory myocardial fibrosis can be reliably identified by CMR imaging with delayed enhancement of the myocardium, which acts as an important predictor of arrhythmias and sudden death in several non-ischemic cardiomyopathies, such as CCC [13]. Couceiro et al., showed for the first-time myocardial injury in patients with acute CD in the Brazilian Amazon [14].

This peculiar epidemiological scenario brings with it the need to investigate the immunological response in patients diagnosed with acute CD through plasma and serum cytokine profiles that can, somehow, be correlated with the degree of cardiac injury in the CMR imaging to improve the care and monitoring of these patients. No study has yet described the inflammatory profile of patients with the disease in its acute form who underwent treatment, nor has it addressed their correlation with the quantification of myocardial injury. It is also important to note that treatment with benznidazole (Rochagan, 5–7 mg/kg for 60 days) is made right after parasitological diagnosis with the thick blood smear technique, and serological tests are used for clinical follow-up. Few studies are available that describe this serological evaluation after treatment in patients with acute CD [15].

Therefore, this study aims to provide an innovative approach to the initial characterization of the immune response in patients with ACD and correlate with the presence of fibrosis in CMR imaging, since many studies that address the expression of these cytokines are limited to CCC and experimental models.

## Methods

### Ethics statement

This study involved the approval of four projects by the Ethics Review Board at Fundação de Medicina Tropical Dr. Heitor Vieira Dourado (Manaus, AM, Brazil) from 2017 to 2022 (CAAE: 69904017.9.0000.0005 - July, 2017/ 66077017.8.0000.0005 – May, 2017/ 33876120.2.0000.0005 – July, 2020 and 53484821.5.0000.0005 – February, 2022). Participants read and signed the written informed consent form before enrollment, in agreement with Resolution 466/12 of the Brazilian National Health Council and ethical guidelines of the 1975 Declaration of Helsinki.

### Study population

This study employed a longitudinal design with patients with ACD from the state of Amazonas and was carried out at the Chagas Disease Outpatient Clinic of Fundação de Medicina Tropical Doutor Heitor Vieira Dourado (FMT-HVD) in Manaus, AM, from 2017 to 2022.

### Patients and sampling

The study population consisted of 35 individuals diagnosed through parasitological and/or serological methods (IgM). Patients who agreed to participate in this study were divided into two groups: (i) pre-treatment – individuals during the acute phase of the disease before benznidazole administration, and (ii) post-treatment – same individuals followed-up with tests performed between 6–12 months after standard treatment (benznidazole 7 mg/kg/day). The control group consisted of 14 healthy individuals with non-reactive serology for CD nor cardiac involvement.

The inclusion criteria consisted of patients aged over 18 years diagnosed with acute CD through direct parasitological and/or IgM serological tests. On the other hand, patients with previous cardiac conditions, any other chronic inflammatory diseases, thyroid dysfunction, hydroeletrolytic disorders, or renal insufficiency were excluded from this study.

### Biological sample collection

Blood samples were collected at the Entomology Unit Nelson Ferreira Fé of the Tropical Medicine Foundation (UENFF/FMT-HVD). A volume of 10 mL was divided into serum and EDTA tubes to separate serum and plasma, respectively (BD Vacutainer, Blood Collection Tubes; Becton, Dickinson and Company, New Jersey, USA). The tubes were then centrifuged at 4000 rpm for 15 minutes. Samples were stored as follows: a volume of 1000 µL of serum and 1000 µL of plasma were stored in microtubes (1.5 ml) at -20ºC.

### xMAP Luminex assay and data

For this study, the interleukins IL-1α, IL-2, IL-4, IL-5, IL-6, IL-10, and IL-17α, as well as TNF-α and IFN-γ were analyzed. The Luminex xMAP (Multiple Analyte Profiling) technology assay, or Luminex assay, is a multiplex method that allows the quantification of several biomarkers at the same time, in a small amount of biological sample. Luminex xMAP technology has a principle similar to the sandwich ELISA, but uses fluorescent-colored microspheres (beads) that bind covalently to capture antibodies. The capture antibodies are placed directly against the desired biomarker. After a series of washes to remove unbound proteins, detection antibodies are added to create the sandwich complex, and then the streptavidin-phycoerythrin conjugate is added.

Essay analysis was performed using the Milliplex Analyst 5.1 software (EMD Millipore). Biomarker concentrations were determined based on standard curve fitting for mean fluorescence intensity (MFI) versus pg/mL. Biomarker levels are expressed in pg/mL or ng/mL according to the standard curve obtained in the assay.

### Cardiac magnetic resonance

Cardiac magnetic resonance imaging (CMRi) was performed before and after treatment using a Philips Ingenia 1.5 T Scanner. The exploration protocol included an initial morphological evaluation, considering bright and black blood gradient echo sequences in the axial and coronal planes. The functional evaluation considers maps of T1 and T2 sequences of cine resonance with the steady-state free precession (SSFP) technique. In addition, a quantitative functional evaluation was performed: structural measurements (diastolic and systolic diameter of the left ventricle, diastolic wall thickness, anterior systolic diameter of the left atrium, left atrial volume in two dimensions, mean lateral diameter of the right ventricle and presence of pericardial effusion) and functional measurements (left ventricular ejection fraction, estimated pulmonary artery pressure and presence and degrees of mitral and tricuspid regurgitation). Tissue characterization was assessed for the presence of myocardial edema using fat-suppressed T2 sequences and the presence of late-gadolinium enhancement (LGE) using 2D and 3D T1 acquisitions. In addition, LGE images were obtained specifying the location and degree of transmurality. The intravenous contrast material used was Gadobutrol (0.1 mmol/kg), a gadolinium-based contrast agent.

### Statistical analyses

Statistical analysis of the data was performed using Stata/MP 14.0 (Statistical Software, College Station, TX: StataCorp LP). Initially, tests were performed to verify data normality using the Shapiro–Wilk test, and demonstrated a parametric distribution. A two-way ANOVA test followed by the Bonferroni post-test was performed for comparisons of soluble molecules concentrations between groups, as well as, Student t test was applied to comparisons between pre- and post-treatment groups. Comparisons of frequencies were performed by Fisher’s exact test. Logistic regression was used for odds ratio estimation of myocardial injury as a predictive method.

For the cytokine network correlation analysis, the Pearson test was applied to the network building, using the Cytoscape 3.10.3 software (Cytoscape Consortium, San Diego, CA, USA). Positive and negative correlations were considered significant when *p*< 0.05. The correlation pattern was used to categorize the strength of correlation as weak (r ≤ 0.35 – thinner line), moderate (r ≥ 0.36 to r ≤ 0.67), or strong (r ≥ 0.68 – thicker line). Dotted lines represented negative correlations and solid lines represented positive ones. The strength of the correlation was represented by the thickness of the lines: the thicker the line, the stronger the correlation. The positive and negative correlations were significant when p < 0.005.

Heatmap clustering was analyzed using Heatmapper (www.heatmapper.ca), for this analysis, we plotted the means of each biomarker across each group. For each row, the means were scaled to mean 0 and standard deviation 1. The dendrogram is generated based on hierarchical clustering with average linkage and 1-Pearson correlation as distance. Statistical significance levels were considered as p < 0.05 and a confidence level of 95%.

## Results

### Population characteristics

**Fig 1:**
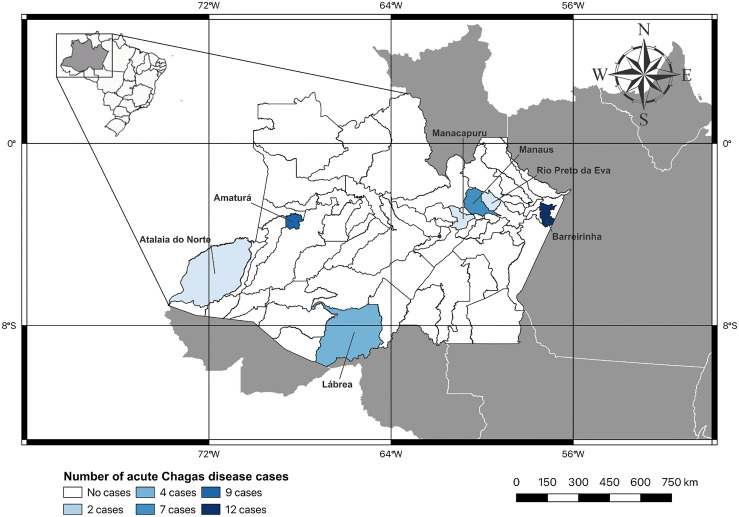
Map of the Amazonas State indicating the number of acute Chagas disease cases in each city. The map was created using QGIS 3.42 software (QGIS, London, UK).

Thirty-five patients with ACD and 14 controls were included between 2017 and 2022, with a mean age of 46.3 ± 17.8 years, mostly male 20 (57.1%), and from three oral transmission outbreaks, with 26 (74.3%) patients (Fig 1).

Basemap shapefile source: https://www.ibge.gov.br/geociencias/organizacao-do-territorio/malhas-territoriais/15774-malhas.html

Upon return, it was possible to recover 25 (71.4%) of the patients, a satisfactory percentage considering the logistical difficulties of transportation in the region. All individuals exhibited clinical features of an infectious syndrome; however, no cardiac symptoms were reported at the time of diagnosis (Table 1).

**Table 1:** Demographic, epidemiological, and clinical data of patients with acute Chagas disease (ACD) and control group.

Variables	ACD	Control group	p-value
	(n = 35)	(n = 14)	
**Age (years), mean (SD), years**	46.3 ± 17.8	45 ± 6.3	0.785
**Sex**			0.160
Male	20 (57.1)	11 (78.6)	
Female	15 (42.9)	3 (21.4)	
**Case Type**			
Outbreak	26 (74.3)	–	
Isolated	9 (25.7)	–	
**Symptoms**			
Febrile syndrome	31 (83.8)	–	
Headache	8 (21.6)	–	
Myalgia/arthralgia	14 (37.8)	–	
Respiratory	4 (10.8)	–	
Cardiovascular	8 (21.6)	–	
Urinary	8 (21.6)	–	
Gastrointestinal	17 (45.9)	–	
Exanthema	1 (2.7)	–	
Edema	6 (16.2)	–	
Inferior limbs	3 (50)	–	
Facial	2 (33.3)	–	
Hands	1 (16.7)	–	
**DTU *T. cruzi***	**n = 31 (88.6)**		
TcIV	29 (93.6)	–	
TcI	2 (6.5)	–	

*Data are described as mean± SD or n (%).*

### Cytokine serum levels and interactions

Elevated levels of all cytokines were observed during the course of acute infection before the start of treatment when compared to levels in the control group, demonstrating an expected effective immune response. When analyzing the concentrations after treatment in the average period of 12 months, it was possible to observe a significant reduction in IL-6 (31.99 ± 30.52 pg/mL vs. 5.23 ± 4.74 pg/mL), TNF-alpha (54.05 ± 46.88 pg/mL vs. 11.23 ± 11.93 pg/mL), IFN-gamma (228.20 ± 363.70 pg/mL vs. 29.41 ± 35.17 pg/mL) and IL-10 (30.46 ± 35.61 pg/mL vs. 9.92 ± 12.71 pg/mL) (Table 2). These differences were significant and demonstrate in humans a classic inflammatory profile in the acute phase and reduction after treatment.

**Table 2:** Cytokine serum levels between control group and ACD groups, before and after treatment.

Groups							
Cytokines levels (pg/mL)	Control (n = 14)	Before treatment (n = 35)	p-value	Control (n = 14)	After treatment (n = 25)	p-value	p-value* before x after
*Pro-inflammatory*							
*IL-1α*	5.47 ± 5.11	6.61 ± 5.94	0.531	5.47 ± 5.11	4.98 ± 4.21	0.751	0.246
*IL-6*	4.36 ± 3.94	31.99 ± 30.52	**0.002**	4.36 ± 3.94	5.23 ± 4.74	0.565	**< 0.001**
*IL-17α*	18.02 ± 15.89	19.09 ± 14.56	0.822	18.02 ± 15.89	16.11 ± 12.01	0.674	0.405
*IFN-γ*	24.24 ± 12.69	228.20 ± 363.70	**0.043**	24.24 ± 12.69	29.41 ± 35.17	0.600	**0.009**
*TNF-α*	8.75 ± 8.65	54.05 ± 46.88	**0.001**	8.75 ± 8.65	11.23 ± 11.93	0.498	**< 0.001**
*Anti-inflammatory*							
*IL-4*	31.6 ± 32.33	42.13 ± 38.59	0.372	31.6 ± 32.33	29.25 ± 29.41	0.819	0.166
*IL-10*	17.97 ± 20.24	30.46 ± 35.61	0.225	17.97 ± 20.24	9.92 ± 12.71	0.135	**0.007**
*Adaptive immunity*							
*IL-2*	18.81 ± 8.99	20.09 ± 9.39	0.667	18.81 ± 8.99	17.67 ± 7.25	0.669	0.288
*IL-5*	6.01 ± 2.92	7.69 ± 6.87	0.385	6.01 ± 2.92	5.98 ± 2.90	0.971	0.246

Data are described as mean± SD. IL: interleukin. IFN: interferon. TNF: tumor necrosis factor. *Overall p-value between acute CD groups.

**Fig 2:**
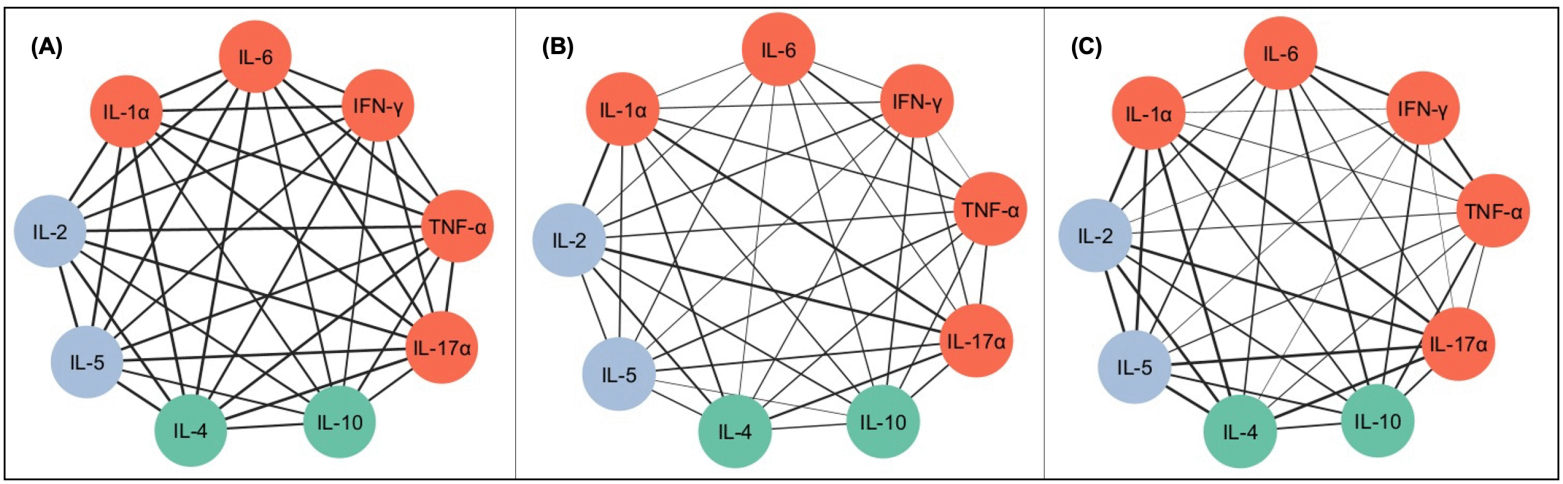
Correlation networks between different serum biomarkers in patients with acute Chagas disease (ACD). (A) Biomarker networks in the control group. (B) Biomarker networks in ACD patients before treatment. (C) Biomarker networks in ACD patients after treatment. Notes: The black continuous lines show positive correlations between the biomarkers. The strength of the interactions was represented by different line styles according to the correlation coefficient (r) ranges. The colors represent the classification of the cytokines: pro-inflammatory (orange), anti-inflammatory (green) and adaptive immunity origin (blue).

In order to understand the interconnection and relationship between the plentiful groups analyzed, correlations analysis was carried out to construct an integrative network of immunological biomarkers. The integrative networks allowed for a panoramic view of data and its association in each group, and the exploratory analysis showed a strong (r ≥ 0.68) positive correlation network for the control group in pro-inflammatory, anti-inflammatory, and adaptive immunity origin cytokines, which indicates a highly coordinated immune profile in the control group (Fig 2A).

On the other hand, in the interactive network of the ACD patients before treatment, was possible to visualize a different interaction profile, highlighting a very strong correlation between IL-2 with IL-1α and IL-17α (r = 0.91 and 0.95, respectively). A presence of several weak correlations was observed (Fig 2B).

Lastly, the interactive biomarkers network displayed for the group of ACD patients after treatment demonstrated a profile similar to the control group, with several moderate or strong correlations between the cytokine groups (Fig 2C). In addition, negative correlations were not observed between biomarkers for any the groups evaluated.

**Fig 3:**
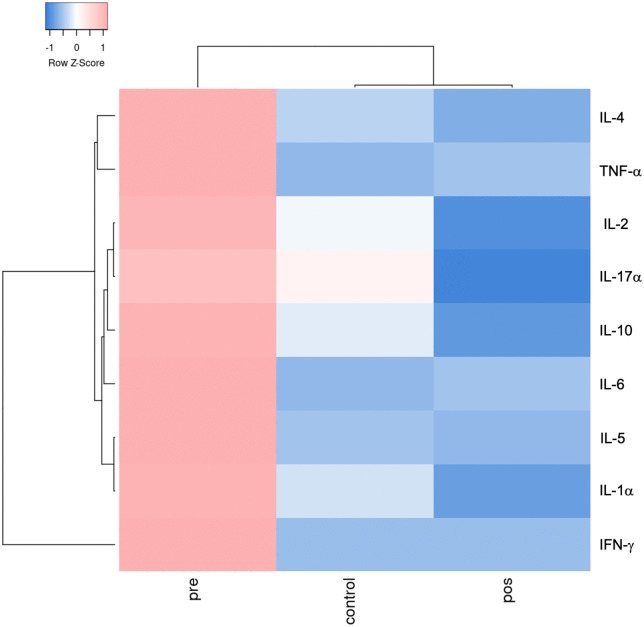
Heatmap clustering analysis of group average concentrations of plasma/serum cytokines. The average plasma/serum cytokines concentrations of CD patients were analyzed using an online heatmapping tool (www.heatmapper.ca, clustering method: Average-linkage).

Heatmaps showed different cytokine expression between patients during the acute phase and after 6–12 month-follow-up. All cytokines’ levels were highly expressed during the onset of the symptoms as expected, but IL-17α and IL-10, as well as IL-2 showed an interesting pattern in patients after treatment. IL-10 levels were lower than the control group, and IL-17α as a regulator cytokine, were lower in the post-treatment group compared to control and the pre-treatment groups. It is expected that IL-17α would mediate immune response. The heatmap demonstrated that the pre-treatment group showed a pattern that is distinct from the other groups. It also demonstrated the post-treatment pattern was similar to controls (Fig 3).

### Cytokine serum levels prediction of reagent serological tests in patients after treatment

When observing the levels of cytokines IFN-γ and TNF-α about the probability of a reactive serology in 2 years after the determination of the markers, it was possible to observe that patients with reactive serologies presented low levels of TNF-α, below the cutoff value of the control group. This profile was not seen in the IFN-γ concentrations (Table 3).

**Table 3:** Cytokine serum levels association with serological 2-year follow-up after treatment (n = 15).

Serology status after treatment				
Cytokines levels during CD onset (pg/mL)	Control (n = 14)	Reactive serology (n = 9)	Non-reactive serology (n = 6)	p-value*
*Pro-inflammatory*				
*IL-1α*	5.47 ± 5.11	5.59 ± 5.12	5.0 ± 4.14	0.819
*IL-6*	4.36 ± 3.94	4.87 ± 4.0	4.89 ± 3.29	0.993
*IL-17α*	18.02 ± 15.89	17.04 ± 13.47	17.18 ± 13.56	0.984
*IFN-γ*	24.24 ± 12.69	23.61 ± 12.44	21.99 ± 11.67	0.804
*TNF-α*	8.75 ± 8.65	8.56 ± 5.57	13.76 ± 12.06	0.275
*Anti-inflammatory*				
*IL-4*	31.6 ± 32.33	31.87 ± 33.03	30.66 ± 32.11	0.945
*IL-10*	17.97 ± 20.24	8.32 ± 9.66	11.0 ± 16.75	0.700
*Adaptive immunity*				
*IL-2*	18.81 ± 8.99	18.77 ± 8.71	17.44 ± 7.42	0.764
*IL-5*	6.01 ± 2.92	6.12 ± 3.43	6.31 ± 3.22	0.915

Data are described as mean± SD. IL: interleukin. IFN: interferon. TNF: tumor necrosis factor. *p-value for comparing reactive and non-reactive serologies.

In addition, IL-10 presented serum levels below the control group when evaluated after treatment, a pattern observed in patients with Chagas cardiomyopathy. The serology was reactive in those who had dosages well below the control group (17.9 pg/ml x 8.3 pg/ml). Patients with inconclusive serology presented profiles very similar to those who had a reactive result.

A correlation network was applied to better understand the interconnection and relationship between the group of cytokines and a reagent serology. During the acute phase (before treatment), a complex interaction was observed, highlighting that almost all cytokines had a moderate to strong correlation, except for IL-6 and IL-1α, IL-2, IL-4 and IL-5 (weak correlations: r = 0.12, 0.21, 0.21 and 0.02, respectively). On the other hand, the post-treatment group lost this complexity and all groups had strong to perfect positive correlations.

### Cytokine serum levels correlation with LV mass on magnetic resonance

Cardiac magnetic resonance imaging was performed in 22/35 (62.9%) of the individuals in the pre-treatment group and 15/25 (60%) of the post-treatment group to detect myocardial injury by the presence of late enhancement. Thus, a prevalence of late enhancement of 27.3% (n = 6) and 33.3% (n = 5) was observed, respectively. Likewise, the percentage of LGE (% of LV mass) was evaluated and no signs of heart failure was observed.

**Fig 4:**
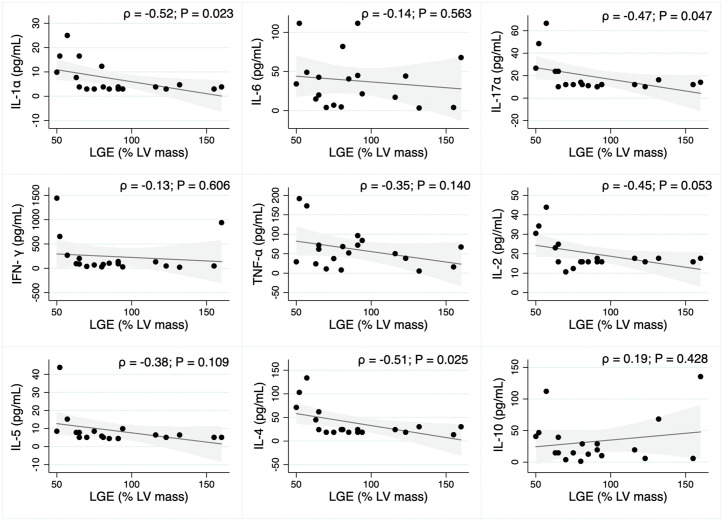
Pearson correlation (ρ) between LGE (% LV mass) and serum levels of cytokines in the pre-treatment group. Correlation strength: weak (ρ ≤ 0.35); moderate (ρ between 0.36 and 0.67); strong (ρ ≥ 0.68).

In the pre-treatment period, a predominant negative correlation was observed between the measured cytokines and the percentage of LV mass of LGE reported on cardiac magnetic resonance imaging. IL-1α, IL-17α and IL-4 showed a moderate (0.36 to 0.67) significant correlation with the presence of LGE. The exception was a positive correlation with IL-10, an important anti-inflammatory biomarker, but without statistical significance (ρ = 0.19, p-value: 0. 428) (Fig 4).

**Fig 5:**
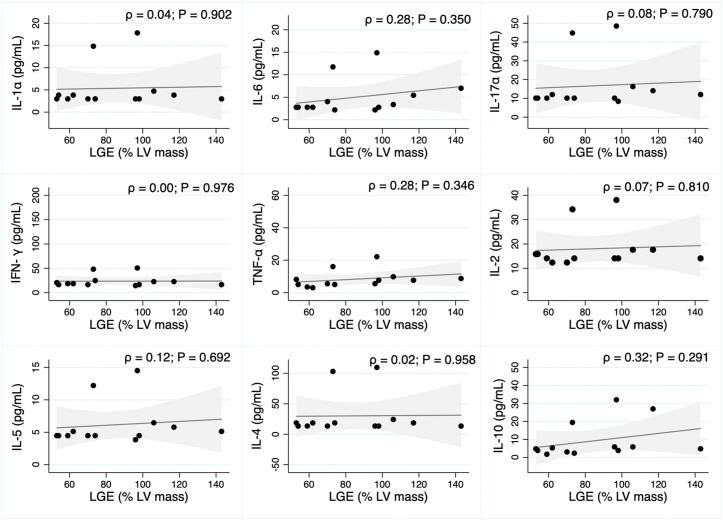
Pearson correlation (ρ) between LGE (% LV mass) and serum levels of cytokines in the post-treatment group. Correlation strength: weak (ρ ≤ 0.35); moderate (ρ between 0.36 and 0.67); strong (ρ ≥ 0.68).

Regarding the post-treatment period, it is possible to observe a distinct pattern in which there is a predominance of positive correlations, even if weak (IL-6, TNF-α and IL-10) or even absence of correlation, regardless of the classification of the cytokines evaluated. No statistical difference was observed (Fig 5).

## Discussion

This is a pioneer study of cytokine profile in patients with ACD from the Brazilian Amazon, specifically from the state of Amazonas. Previous studies had focused on experimental models for ACD or studies with indeterminate and cardiac patients. Therefore, we present an innovative view of what is expected in the immune response of this specific group of patients and hypothesize based on this data about the evolution of the disease even after treatment.

Acute cases in the Brazilian Amazon region have been reported since 1968 in Belém, Pará [16] and several outbreaks have been described in the literature [15,17–19]. In Amazonas, 11 outbreaks have been reported in 10 cities [3], in one of them *T. cruzi* was detected alive in the açaí juice associated with the outbreak in Lábrea (7° 15′ 32″ S, 64° 47′ 52″ W) [20], also several studies showed cardiac alterations in ACD patients [21,22] with 30% prevalence and one case report of a patient that after 5 years developed severe cardiac dysfunction characterized as CCC [23].

### Cytokine serum levels and interactions

Chagas disease immune response has been widely studied, showing patterns in cytokine expression either in humans [24–28] – patients with indeterminate and cardiac forms of the disease – or in experimental models with all different phases, including the acute form [29,30].

Most of the studies until now have reached a common conclusion that divides the cytokine expression into three classic patterns: (1) indeterminate form presenting high levels of IL-10, and low levels of IFN-γ and TNF-α; (2) chagasic cardiomyopathy, on the contrary, with high levels of IFN-γ, TNF-α and IL-6 plus low IL-10 and TGF-β concentrations; (3) worst prognosis related to high concentrations of IL-6 and TNF-α [25,31–33].

High levels of TNF-α and IL-6 in some of the acute patients, different than what we have seen in our cohort, with the highest serum level of IFN-γ, this could have been influenced by the population tested (adults *vs.* children) [25,34]. Higher production and secretion of IFN-γ suggest a relationship with morbidity in Chagas disease, although our results cannot make this kind of assumption, we raise a concern about long-term follow-up, considering that in the after-treatment group the highest serum level was still of IFN-γ when compared to the pre-treatment group (p = 0.011) [24,35].

For acute infection, experimental models of Chagas disease in mice have shown a strong T-helper-1 immune response with CD4 and CD8 cells. This response is characterized by the production of IFN-γ, TNF-α, and IL-12 [32,36,37]. Macrophages, activated by TNF-α and IFN-γ, and CD8 cells are crucial in the control of acute infection [38]. To sum up, this model also showed an increased Th1 cytokine profile while Th2 cytokines are suppressed [39].

Barreto-Albuquerque *et al.* 2018 tested two different routes of acute infection: oral *vs.* gastrointestinal, which showed higher concentrations of IFN-γ, IL-17, and TNF-α, IFN-γ and IL-17 coincided with high levels for oral transmission. The highest level was for IL4, an important marker of Th2 activation, with anti-inflammatory activity and a special role in preventing unwanted excessive tissue inflammation [29,40].

On the other hand, exacerbated IFN-γ production favors a strong Th1 response [36]. Out of all the cytokine panel measured in this cohort, IL-4 was the second highest level identified in the after-treatment group with no statistical significance (p = 0.479).

In a clinical study, a predominance of CD4 + /HLA-DR low/- monocytes in indeterminate patients suggests an immunoregulatory role that could delay the evolution to CCC [40,41], on the other hand, CD8 + T-lymphocytes might be important effector cells in parasite control [40,42]. Another study has reported that IFN-γ declines as early as 12 months after therapy and it became undetectable in almost 50% of treated patients [36,43]. In our cohort, decreased IFN-γ serum concentration, as close to the control group, was observed (p = 0.011).

A further study regarding IL-10, another important marker of evolution, it presented lower levels in the post-treatment period below the control, including similar concentration values (9.92 ± 12.71 vs. 9.93 [2.31-25.58]) to a previous study in the group of patients with Chagas cardiomyopathy by Sousa *et al.*, 2014 [7]. IL-10 plays an important role during acute infection as a crucial anti-inflammatory cytokine, regulating immune response, limiting inflammation and relating to long-term effects of etiological treatment with benznidazole [29].

Some studies have shown that regulatory cytokines such as IL-17 are crucial for protective immune response, where high serum or plasma levels were associated with less parasite load^2733^. Bestetti et al., 2019 studied patients with CCC and found that this population had a higher level of immunomodulation [26]. In our study, we did not find a significant difference in IL-17 levels before and after treatment.

Low concentration of IL-12 was associated with the presence of Chagas cardiomyopathy [44]. However, our results did not show a profile that could be used in the future as a marker. The expected increase in the acute phase was followed by a decrease after etiological treatment and stabilization to near normal levels.

An extensive analysis of biomarkers in Chagas cardiomyopathy was conducted in 2015 by Keating *et al*. [45], the study tested 22 biomarker signatures to classify *T. cruzi* seropositive subjects into clinical CD groups and showed a different pattern of the cardiac group compared to blood donors/controls and non-cardiac groups.

Although this work focused on the severe form of Chagas disease, our study, for instance, focused on describing the serum biomarker levels in acute Chagas disease patients who underwent etiological treatment with benznidazole (Rochagan). We found a different pattern between patients during the acute phase and after 6–12 month-follow-up. IL-10 levels were lower in the post-treatment than in the control group, and in very similar concentrations for cardiomyopathy patients [7].

### Cytokine serum levels prediction of reagent serological tests in patients after treatment

It is well known that the indeterminate form of Chagas disease is prevalent in approximately 70% of patients, and serodiscordant subjects often display a simultaneous production of IFN-γ and IL-2 in response to *T. cruzi* antigens [2,35]. A hypothesis was raised about a possible correlation between some cytokines in patients with reagent *vs.* non-reagent serology. We did not find any important interactive connection in the post-treatment group that would justify a reagent serology in this group of patients.

The levels of TNF-α and IFN-γ had an expected increase, with a reduction in the post-treatment group down near the control group level, which is in line with previous studies that associate these low levels of TNF-α with an indicator of the indeterminate chronic disease [33]. In our study, it was possible to observe that these lower TNF-α levels were expressed in patients with reactive serology, but without any statistical significance.

Factors that lead indeterminate patients to develop CCC are still unknown, as well as, is obscure how patients from the acute phase could develop cardiac alterations during the course of the disease even years after specific treatment. Our study tried to show that serological parameters when compared to the cytokine expression have some degree of correlation, no difference was observed.

### Cytokine serum levels correlation with LV mass on magnetic resonance

Cardiac magnetic resonance with delayed enhancement is the gold standard for myocardial fibrosis quantification, and is well established to safely identify post-inflammatory myocardial injury, an important arrhythmia, and a sudden death predictor in nonischemic cardiomyopathies, including chronic Chagas cardiomyopathy [46]. In the Brazilian Amazon, Couceiro *et al.* 2022 showed 58.3% and 50% of myocardial injury in acute Chagas disease patients before and after 12-month follow-up, respectively [14].

In our study, we demonstrated 27.3% of myocardial injury in the acute phase, as well as a moderate negative correlation with IL-1α, IL-17α, and IL-4, which was statistically significant. For the acute phase after treatment, 33.3% showed a myocardial injury and a different pattern with a predominance of positive correlations; in addition, no statistical difference was found. It is important to note that IL-10, as an anti-inflammatory cytokine, plays a key role in protective immune response and better cardiac function (if high levels), showed the same profile and similar correlation coefficient in both groups, despite the lack of statistical significance.

In addition to the clinical improvement observed in the post-treatment period, previous studies have also shown a favorable evolution in imaging exams such as magnetic resonance imaging, reinforcing the benefits of therapeutic intervention in the acute phase [14,22]. In the present study, the return of cytokine levels to standards similar to those of the control group after treatment can be interpreted as an indication of efficacy in modulating inflammatory response.

These findings suggest that, in addition to clinical recovery, there is a normalization of the immune profile, which corroborates the importance of early and adequate interventions in the management of the condition.

### Study limitations

Although this study provides an innovative overview of the cytokine profile in patients with ACD, certain limitations must be acknowledged. These include the small sample size, which was constrained by the spontaneous demand for acute cases in the region, and the logistical challenges in conducting follow-up after treatment. Additionally, this study did not focus on nutritional status, which may have limited some interpretations due to the risk of potential biases.

## Conclusion

In this cohort of autochthonous patients from the Brazilian Amazon, we characterized the serum cytokine profile at the onset of acute symptoms and 6–12 months post-etiological treatment, which appears to promote a systemic reduction in both pro-inflammatory and regulatory cytokines.

While the negative correlation between specific cytokines and LGE suggests a potential link between the immune response and the degree of myocardial injury, these findings remain exploratory due to the limited sample size and should be interpreted with caution.

Lastly, this pilot work is valuable to improve knowledge of ACD scenario in our state as well as being vital for upcoming studies in the Brazilian Amazon to better understand and unravel the immune response in patients with acute Chagas disease, allied to the disease’s long-term progression after treatment.
